# Increasing Natural Killer Cell Activity of Mineral Nanomaterial ALP1018 in Healthy Adults: A Randomized, Double-Blind, Placebo Comparative Clinical Trial

**DOI:** 10.3390/nu16060850

**Published:** 2024-03-15

**Authors:** Soon-Ae Kim, Seyl Kim, Hyungyung Chai, Junlae Cho, Yu-Jin Paek

**Affiliations:** 1 Department of Pharmacology, School of Medicine, Eulji University, Daejeon 34824, Republic of Korea; 2 Department of Nanotech Research and Development, Ferramed Inc., Daejeon 34141, Republic of Korea; kimseyl@ferramedi.com (S.K.); chojl@ferramedi.com (J.C.); 3 Research Institute, MediCRO Co., Ltd., Anyang 14067, Republic of Korea; biostat@medicro.co.kr; 4 School of Pharmacy, University of Sydny, Sydney 2000, Australia; 5 Department of Family Medicine, Hallym University Sacred Heart Hospital, Anyang 14068, Republic of Korea

**Keywords:** ALP1018, immune, clinical trial, natural killer cell, cytokine

## Abstract

This randomized, double-blind, placebo comparative clinical trial aimed to determine the immune-enhancing effects and safety of a nanomaterial with iron and zinc (ALP1018) in healthy adults. Participants who met the inclusion criteria were recruited for this study (*n* = 80) and randomly assigned to either the test group (*n* = 40), which was given Alp1018 in capsule form, or the placebo group (*n* = 40), which was given crystal cellulose capsules of identical appearance, weight, and flavor for 8 weeks. Compared to baseline, natural killer (NK) cell activity (%) increased in the test group after 8 weeks, although there were no changes in the placebo group. Furthermore, in the subgroup analysis of Coronavirus disease 2019 (COVID-19) affected participants, significantly increased NK cell activity was observed in the test group at 4 (*p* < 0.05) and 8 weeks (*p* < 0.05). No significant differences were observed in cytokine levels between the two groups. ALP1018 supplementation appeared to enhance immune function by improving NK cell activity without adverse effects in healthy adults.

## 1. Introduction

In an era marked by rapid industrialization, environmental challenges, and an aging population, humans face a myriad of diseases that remain unresolved through advanced science and medical technology. Modern lifestyles, characterized by stress, dietary imbalances, and environmental alterations, have led to increased exposure to health risks, including lifestyle diseases and stress-related disorders. The immune system, a complex network involving various cells and organs, is essential for defense against a range of external threats. Consequently, interest in enhancing immune function to counteract the threats posed by pollutants, pathogens, and infectious agents has increased. This comprises innate immunity, which provides rapid, non-specific defense, and adaptive immunity, offering specific responses via T and B cells. Macrophages, which are key components of the immune system, play a critical role in both innate and adaptive responses by secreting essential cytokines and interleukins [1]. In addition, the presence of natural killer (NK) cells is essential for maintaining a basal level of phagocytic activity and targeting aberrant cells such as virally infected and tumorigenic cells [2,3]. NK cells are primarily recognized for their cytotoxic capabilities and cytokine production upon activation [4]. Regarding their cytotoxicity, they specifically target cells that are infected, transformed, or under stress. Through this mechanism, NK cells play a crucial role not only in eliminating deleterious cells but also in maintaining cellular homeostasis [5]. Traditionally, the bone marrow has been identified as the principal site for NK cell generation, where interactions with other cellular entities, cytokines, and soluble factors facilitate and promote the maturation of NK cells [6].

Enhancing human immune function through dietary and supplemental interventions has become increasingly important in contemporary healthcare research. Iron is essential for many physiological processes in the body, including erythropoiesis, immune function, and host defense, as well as essential cellular activities such as DNA replication and repair, mitochondrial functions including oxidative phosphorylation and enzymatic reactions that require iron as a cofactor [7]. Zinc is a cofactor involved in various enzymatic reactions necessary for immune function, including DNA replication and repair, RNA transcription, cell division, and activation. It also contributes to the normal function of proteins involved in gene expression [8]. Furthermore, both iron and zinc are crucial for the development and proper functioning of immune cells. Therefore, adequate levels of iron and zinc are essential for maintaining a healthy immune system and supplements may be beneficial in cases of deficiency. However, excessive intake of these minerals can have adverse effects; therefore, supplementation should be administered judiciously. ALP1018, a polymeric compound comprising iron and zinc in a balanced ratio, has emerged as a potential immune supplement. Preclinical toxicological tests have indicated no mutagenic or genotoxic potential [9]. In response to the increasing prevalence of upper respiratory tract infections and the need for effective immune support, this study investigated the potential benefits of ALP1018 in healthy adults. This study details an 8-week randomized, double-blind, placebo-controlled human clinical trial designed to rigorously evaluate the efficacy and safety of ALP1018 in enhancing immune function, particularly NK cell activity.

## 2. Materials and Methods

### 2.1. Trial Supplements

ALP1018 (CAS Registry Number: 1254036-30-0) was supplied by the manufacturer (Hylot Korea Inc., Daejon, Republic of Korea) in the appearance of a pale yellow-green powder [9]. ALP1018 and its placebo control were manufactured and packaged at 500 mg/capsule by the requester of the trial and provided to the clinical trial institution. The trial supplements were ensured to be identical in appearance and characteristics, with no discernible differences to the naked eye, and the weight difference was kept minimal. Additionally, the same label was attached to the supplements for the human clinical trial to maintain blinding (blind) for both the participants and testers.

### 2.2. Participants

This study enrolled healthy men and women aged 19–80 years who had experienced more than two episodes of upper respiratory infection symptoms within the past year. Eighty participants (forty per group) were randomly assigned to either the test or placebo group. Participants were selected based on voluntary consent and compliance with the inclusion and exclusion criteria outlined in the human clinical trial protocol (Table 1).

### 2.3. Study Design, Screening, and Randomization

This clinical trial was conducted until 31 July 2023, following approval from the Hallym University Sacred Heart Hospital’s Biomedical Research Ethics Committee (IRB). The participants underwent screening tests (Visit 1/Visit 2) according to the clinical trial protocol to evaluate their eligibility based on the inclusion and exclusion criteria. Following a successful screening and baseline visit (Visit 2), the participants were randomized 1:1 into either the test or placebo groups. Participants were instructed to take one capsule of the trial supplement daily for 8 weeks, starting from the day of randomization. All participants were required to visit the clinical trial institution twice during the intervention period (at 4 [Visit 3] and 8 weeks [Visit 4]) for scheduled assessments. These visits included evaluation of vital signs, laboratory tests for safety assessment, and functional evaluations.

### 2.4. Evaluation of Diet and Physical Activity

Dietary intake was assessed in participants in the Per Protocol (PP) group who completed the clinical trial schedule without any major protocol violations. Nutrient intake during the trial period was evaluated based on the dietary records maintained by the participants. The dietary diary from the previous day was collected and analyzed using CAN-Pro 4.0^®^, a computer-aided nutritional analysis program of the Korean Nutrition Society Forum, Seoul, Republic of Korea, and average values were calculated. Additionally, exercise logs, which included the type of physical activity, duration, and frequency, were used to analyze the amount of energy expended by the participants through exercise. This comprehensive approach allowed for a detailed assessment of both caloric intake from food and caloric expenditure through physical activity, providing a holistic view of the participants’ nutritional and exercise habits.

### 2.5. Outcome Measures

#### 2.5.1. NK Cell Activity

NK Cell activity was assessed by isolating NK cells (Mojosort™ Human NK cell isolation Kit, BioLegend, San Diego, CA, USA) from pre-isolated peripheral blood monocytes (Lymphoprep™, StemCell, Vancouver, BC, Canada) in whole blood samples. To evaluate the activity of NK cells, an LDH calorimetric cytotoxicity assay (CytoTox 96^®^ Non-Radioactive Cytotoxicity Assay, Promega, Madison, WI, USA) was performed using NK cells isolated from blood samples of the participants and the K562 cell line, which serves as a target cell type representative of cancer cells. The NK cells were co-cultured with K562 cells at predetermined effector-to-target (E:T) ratios (12.5:1, 25:1, 50:1). LDH release assays were conducted using ELISA reader (Sunrise^TM^, TECAN, Männedorf, Switzerland) and measuring absorbance at 492 nm.

#### 2.5.2. Cytokine Levels

Blood samples were collected from participants during their baseline visit (Visit 2) and from the third and fourth visits. These samples were processed to obtain serum, which was stored frozen until further analysis. Cytokine levels were quantified using the MILLIPLEX MAP Human Cytokine/Chemokine Magnetic Bead Panel, focusing on Interferon-γ (IFN-γ), Tumor necrosis factor-α (TNF-α), and Interleukin-6 (IL-6) (Cat. No HCYTMAG-60K, MERCK, Darmstadt, Germany). Fluorescence detection was conducted using the MAGPIX^®^ System (Luminex-Merck, Darmstadt, Germany). The acquired data were analyzed utilizing xPONENT software 4.3.229.0 and cytokine concentrations were extrapolated from the standard curves.

### 2.6. Safety Outcome Measurements

A safety evaluation of the human clinical trial was conducted on participants who had consumed the trial food at least once and included adverse reaction monitoring, diagnostic laboratory tests, vital signs, electrocardiography, and physical examination. For diagnostic laboratory tests, participants were instructed to fast for more than 12 h before visiting the clinic. If screening visit test results from within the past 2 weeks were available (excluding white blood cell [WBC] and pregnancy tests), they were applied. The investigator had the discretion to order retests for any abnormal results. However, the diagnostic tests during the screening visit were conducted only in participants who met the study selection criteria. The testing included hematological tests, comprising WBC count, red blood cell (RBC) count, hemoglobin, hematocrit, and platelet count, whereas blood biochemistry tests comprised total bilirubin, alkaline phosphatase (ALP), gamma-glutamyl transferase (gamma-GT), alanine aminotransferase (ALT), aspartate aminotransferase (AST), glucose, total protein, albumin, blood urea nitrogen (BUN), creatinine, and creatine kinase (CK).

### 2.7. Statistical Analysis

Statistical analyses were performed using SAS^®^ version 9.4 (SAS Institute, Cary, NC, USA). In instances of participant dropout or other missing data, the Last Observation Carried Forward method was used to maintain the integrity of the dataset. Continuous variables are represented as mean ± standard deviation (SD), whereas categorical variables are expressed as numbers and percentages. Both Full Analysis Set and PP approaches were used to evaluate the final results. To assess the differences between the ALP1018 and placebo groups, independent *t*-tests were performed on continuous variables measured at baseline and on the mean changes in primary and secondary functional evaluation parameters before and after intake within each group. In cases in which the data did not conform to a normal distribution, non-parametric tests were used as an alternative analytical method. Furthermore, items with heterogeneous baseline and demographic variables were adjusted as covariates in an Analysis of Covariance for analysis. This approach was used to ensure a more accurate interpretation of the data by controlling for potential confounding variables. Throughout the analysis, a *p*-value < 0.05 was considered indicative of statistical significance, adhering to the conventional criteria for inferential statistics in clinical research.

## 3. Results

### 3.1. Participant Demographic Characteristics

**Figure 1:**
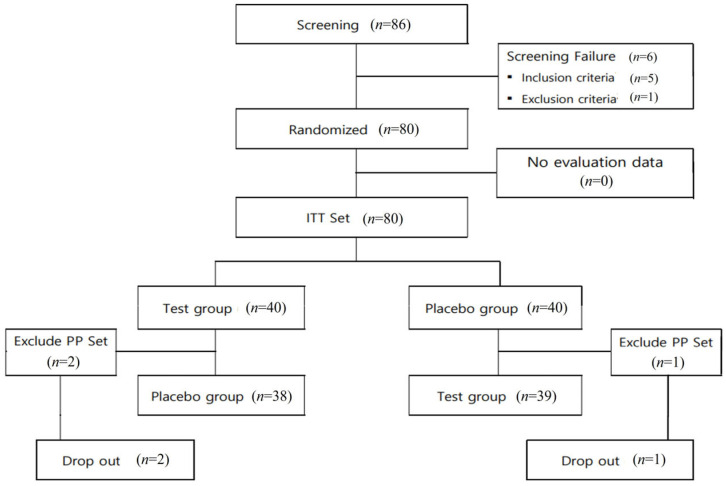
Flow chart of study participants.

After screening 86 volunteers, 80 were selected (21 men, 59 women) as eligible candidates for the intention-to-treatment set and randomly assigned to the test or placebo groups (*n* = 40 in each group). Primary analysis for functional evaluation was conducted using the PP set. During the course of the study, three participants withdrew their consent, resulting in dropout (two and one from the test and placebo groups, respectively). Consequently, 77 participants (38 and 39 in the test and placebo groups, respectively) completed all the procedures specified in the human clinical trial protocol. This subset of participants formed the basis of the functional evaluation analysis presented in this study (Figure 1). Participants in this human clinical trial were selected based on their voluntary consent and after assessing their eligibility according to the inclusion and exclusion criteria specified in the clinical trial protocol. This process involved conducting screening tests (Visits 1 and 2). Following the successful completion of the baseline visit procedures (Visit 2), eligible participants were randomly assigned in a 1:1 ratio to either the test or placebo group.

In the investigation of medical history related to COVID-19 and upper respiratory infections (URI), such as colds, tonsillitis, pharyngitis, laryngitis, sinusitis, otitis media, and rhinitis among the 80 study participants, in the test group and placebo group, 32 and 29 people, respectively, were found to have a history of COVID-19. None of the patients reported any post-COVID sequelae. Additionally, all 80 participants experienced more than two episodes of URI symptoms within the year preceding the screening. WBC counts for all participants, measured during the screening tests, were within the range of 3–8 × 10^3^ cells/µL, meeting the inclusion criteria for this clinical trial. No significant differences in WBC counts were observed between the intake groups (*p* > 0.05). In addition, no significant differences were observed in age, sex, BMI, vital signs, smoking status, or drinking status between the two groups (Table 2).

### 3.2. Trial Procedure and Follow-Up

From the day of randomization, the participants were instructed to take one capsule of the trial supplement daily for 8 weeks. To evaluate the efficacy and safety of the trial supplements, all enrolled participants were required to visit the clinical trial institution twice during the 8-week period, specifically at the 4- (Visit 1) and 8-week (Visit 2) marks. These scheduled visits were designed to assess both the efficacy and safety of the trial food, ensuring comprehensive monitoring of the responses of the participants to the intervention. This structured approach allowed for a thorough evaluation of the effects of the trial supplements within a controlled and systematic framework. Throughout the human clinical trial, both the test and placebo groups demonstrated a compliance rate of >90% with the prescribed intake of trial food. No significant differences were observed in supplementation compliance between the test and placebo groups (Table S1).

### 3.3. Dietary Intake and Physical Activity Evaluation

Over the course of the human clinical trial, no statistically significant differences were observed in nutrient intake between the test and placebo groups (Table S2). This was also true for iron and zinc, which are the main components of ALP1018. Considering that the nutrient intake levels for both groups were within the recommended daily intake range for adults (average age 40.18 ± 9.90 years; Iron: 10–14 g, Zinc: 8–11 mg), it was inferred that the dietary intake had a more significant impact than the intake of the trial food. Analysis of the recorded physical activity logs of the participants revealed no statistically significant differences in the activity levels between the two groups during the trial (Table S3).

### 3.4. Efficacy and Safety Evaluation

#### 3.4.1. NK Cell Activity

**Figure 2:**
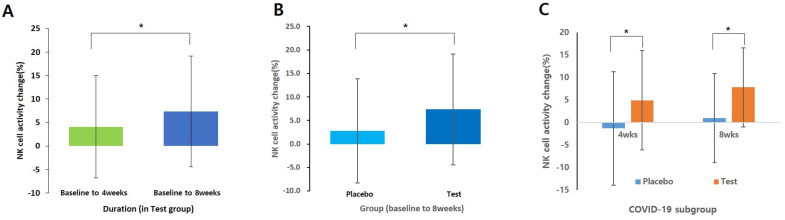
Changes in NK cell activity (%) in the Test group and placebo group (E:T ratios = 50:1): (**A**) Analyzed using a paired *t*-test (baseline vs. 8 weeks in test group); (**B**) Analysis using independent *t*-test only baseline to 8weeks data; (**C**) Analysis using independent *t*-test only COVID-19 subgroup. Data shown mean ± standard deviation. * *p* < 0.05.

NK cell activity at ratios of 12.5:1, 25:1, and 50:1 was measured at baseline (Visit 2), third visit (after 4 weeks of intake), and fourth visit (after 8 weeks of intake) to compare changes over the 8-week period. The results showed a statistically significant increase in NK cell activity (50:1 ratio) in the test group compared to that in the placebo group after 8 weeks of intake (*p* = 0.048; Figure 2A). Additionally, a significant increase in the average NK cell activity was observed in the test group after 8 weeks of intake compared to the baseline (*p* = 0.026; Figure 2B), whereas no significant change was found in the placebo group (*p* > 0.05). However, no statistically significant differences were observed in NK cell activity at ratios of 12.5:1 and 25:1 between the groups, or before and after intake (*p* > 0.05). A subgroup analysis focusing solely on participants with a history of COVID-19 infection revealed that the test group showed a statistically significant increase in NK cell activity changes (50:1 ratio) compared with the placebo group (4 weeks *p* = 0.039, 8 weeks *p* = 0.011; Figure 2C). Furthermore, the analysis of the mean change in NK cell activity before and after intake indicated a significant increase in the test group after 8 weeks (*p* = 0.001), whereas no significant change was observed in the placebo group.

#### 3.4.2. Cytokine Levels

Significant differences were not observed in IL-6, TNF-α, and IFN-γ levels between the two groups (Table S4).

#### 3.4.3. Safety Outcome Monitoring

Throughout the study, the participants exhibited no significant alterations or discrepancies in safety indicators, including electrocardiograms and vital signs, specifically blood pressure and pulse rate. All laboratory parameters remained within normal limits, and no side effects were observed, confirming the safety profile of the intervention. Although RBC count, hematocrit, and serum albumin levels significantly increased in the test group, other diagnostic laboratory analysis results were not significantly different between the two groups (Table S5).

## 4. Discussion

NK cells, a key component of white blood lymphocytes within the innate immune system, play a pivotal role in mediating immune responses against infections such as viruses, bacteria, or parasites, as well as in cancer immunity [10]. NK cells, known for secreting cytokines like IFN-γ, have the unique ability to simultaneously activate macrophages and T cells [11]. Unlike T and B cells, which rely on specific antigen receptors, NK cell activation is regulated by a complex combination of various activating and inhibitory receptors on their cell surface [12]. These cells play a crucial role in immune surveillance against tumors induced by carcinogens or naturally occurring cancers, as they can destroy tumor or virus-infected cells without the need for antibodies [13]. The activation of NK cells is known to enhance immunity, and their decreased activity or absence can be indicative of disease, serving as a predictive marker for assessing immune function and preventing complications such as respiratory infections during treatment [14]. Initial clinical studies in humans have revealed that individuals with healthy lifestyles exhibit higher NK cell activity. Therefore, in this study, increased NK cell activity in the test group administered ALP1018 suggests the potential for an enhanced immune response in the general adult population [15,16,17].

NK cells have been implicated in >50 out of more than 400 primary immunodeficiency diseases (PIDs). Specifically, several PID cases exhibit direct abnormalities in NK cells, involving mutations in genes such as *GATA2*, *MCM4*, *MCM10*, *IRF8*, and *FCGR3A*. These mutations can affect NK cell numbers, subsets, and functions leading to reduced NK cell counts.

Various environmental cues initiate a complex network of transcription factors during their early development, one of which is GATA2, a master regulator that drives the commitment of common lymphoid progenitors into immature NK progenitors [18]. It is suggested that the serine/threonine-rich domain of human MCM4 is important for DNA replication and genome integrity [19]. Mace et al. (2020) highlight that MCM10 deficiency, through studies in primary fibroblasts and NK cell lines, disrupts cell cycle progression and activates DNA damage-response pathways [20]. IRF8 is essential for the development and functional maturation of human NK cells, with its dysregulation linked to severe human diseases, underscoring its vital role in antiviral defense [21]. The FCGR3A gene encodes CD16, a low-affinity receptor (50–70 kDa) that binds to the Fc region of IgG. *FCGR3A* mutations, on the other hand, impair NK cell function despite normal cell counts, due to a mutation affecting the CD16 receptor. Patients with such mutations experience compromised natural cytotoxicity; however, they retain Antibody-Dependent Cellular Cytotoxicity [22,23]. NK cells are crucial in the response to acute viral infections, such as those caused by flaviviruses and influenza. Clinically, NK cell deficiencies are associated with heightened susceptibility to herpes virus infections, such as Epstein-Barr virus, herpes simplex, and varicella zoster, which are responsible for diseases in approximately 60% of NK cell deficiency cases. Approximately half of the patients with reported NK cell deficiencies experience premature death, emphasizing the severity of these conditions [24,25].

NK cells kill virus-infected cells through two primary mechanisms: engaging extracellular death receptors on target cells, notably the Fas ligand (FasL) and tumor necrosis factor-related apoptosis-inducing ligand (TRAIL), releasing cytolytic granules containing perforin, and granzymes. Viruses such as cytomegalovirus and encephalomyocarditis virus can trigger the expression of death receptors on infected cells, which then interact with FasL and TRAIL on NK cells, leading to apoptosis. The granules released by NK cells induce cell death in target cells through caspase-mediated signaling pathways. In addition to direct cytotoxicity, NK cells contribute to the antiviral defense by releasing a variety of proinflammatory cytokines. The activation and subsequent action of NK cells are determined by the balance between activating and inhibitory receptor engagements, which are influenced by the presence of specific cytokines [26,27,28,29].

In late 2019, the zoonotic viral pathogen SARS-CoV-2 emerged, leading to >60 million infections and 1.5 million deaths in the first year of the pandemic, causing COVID-19, a respiratory and vascular disease that can result in acute respiratory distress syndrome and multi-organ failure in severe cases. Although the complete pathogenesis of COVID-19 remains unclear, a misdirected and hyperactivated immune system, including a significant contribution from NK cells, is considered to play a crucial role in disease severity [30,31]. In severe COVID-19 cases, a notable reduction in NK cell numbers and function has been observed, contributing to the decreased clearance of infected cells and an unchecked rise in inflammation markers. This skewing of the immune response towards an overwhelmingly inflammatory phenotype emphasizes the necessity to restore NK cell effector functions, which could potentially ameliorate the immune imbalance and effectively combat SARS-CoV-2 infection [32,33,34]. In this study, a subgroup analysis was conducted on COVID-19(+) participants, and a significant increase in NK cell activity was observed in the test group. The observed increase in NK cell activity may be helpful in preventing the development of severe COVID-19.

In the context of immunocompetence, deficiencies in essential minerals such as zinc and iron have been shown to affect both humans and experimental animals. Research indicates that a lack of these nutrients can increase the susceptibility to infectious diseases. Various studies have highlighted alterations in specific immune responses due to mineral deficiencies, although the precise immunological roles of zinc and iron in NK cells are yet to be fully identified [35]. Sherman et al. reported that pregnant rats fed diets with different iron levels (6, 10, or 250 ppm) throughout gestation and lactation exhibited marked variations in the immunity of their offspring. After birth, when these pups were subjected to the vaccinia virus, the analysis showed a significant reduction in spleen NK cell activity among those that were iron-deficient [36]. These findings are consistent with those of other studies such as those by Hallquist et al., who reported reduced IFN production and NK cell stimulation due to iron deficiency [37]. Similarly, Spear et al. observed that moderate iron deficiency contributes to cancer development and impairs NK cell cytotoxicity in a carcinogen-induced tumor model. These studies collectively underscore the critical role of iron and other minerals in maintaining robust immune responses and the potential risks posed by their deficiencies [38].

The role of zinc in immune function has been highlighted in the field of nutrition. Using a rat model, Oztürk et al. suggested that zinc deficiency is associated with decreased NK and LPS-activated NK cell activity [39]. Furthermore, in vitro studies have revealed that zinc supplementation enhances the killing activity of primary NK cells sourced from healthy donors. A rapid influx of zinc, particularly after a period of deficiency, significantly boosts NK cell cytotoxicity [40]. Collectively, these findings underscore the critical importance of zinc in regulating and enhancing the functionality of NK cells within the immune system. In this study, we observed noteworthy increases in hematocrit, RBC count, and serum albumin levels among participants in the test group who received ALP1018. Iron supplementation may play a role in supporting liver function and facilitating blood cell production. ALP1018 has been speculated to serve effectively as an iron supplement and shows promise for mitigating the reduction in NK cell activity associated with iron deficiency.

## 5. Conclusions

In conclusion, this study identified the immune-enhancing and hematopoietic effects of ALP1018 in healthy Korean individuals. To obtain a full valuation, it must be verified in groups other than Koreans. Although changes in NK cell activity have been observed, changes in blood cell populations, including immune cells, cytokines, and molecular signaling related to NK cell activity, have not yet been studied. Further in vitro and in vivo studies are required to elucidate the mechanisms underlying this immune activation.

## Figures and Tables

**Table 1:** Inclusion and exclusion criteria.

Inclusion Criteria
(1) Adults aged 19–80 years at the time of screening. (2) Individuals with a white blood cell count between 3 × 10^3^ cells/μL and 8 × 10^3^ cells/μL during the screening test. (3) Individuals who have had two or more upper respiratory infections (such as colds, tonsillitis, pharyngitis, laryngitis, sinusitis, otitis media, rhinitis, etc.) within the year prior to screening. (4) Individuals who understand and are capable of complying with the test methods and procedures outlined in the human clinical trial protocol. (5) Individuals who voluntarily agree to participate in the human clinical trial and have signed the informed consent form (ICF) prior to any screening procedures.
**Exclusion Criteria**
(1) Individuals with immune-related diseases (leukemia, autoimmune diseases, acquired immunodeficiency, systemic lupus erythematosus, Sjögren’s syndrome, etc.). (2) Individuals with clinically significant acute or chronic cardiovascular and cerebrovascular diseases (congestive heart failure, coronary artery disease, myocardial infarction, etc.), endocrine disorders (uncontrolled hyperlipidemia, thyroid diseases), respiratory disorders (asthma, etc.), hepato-biliary system, renal and urological, neurological (central or peripheral), psychiatric, musculoskeletal, inflammatory and hematologic-oncologic, gastrointestinal diseases requiring treatment. (3) Individuals who have received vaccinations (influenza, pertussis, measles, chickenpox, tetanus, shingles, pneumonia, etc.) within the last month as per screening visit. (4) Individuals with a body mass index (BMI) below 18.5 mg/m^2^ or above 30.0 mg/m^2^ at the time of screening visit. (5) Individuals who have been vaccinated for COVID-19 within the last three months as per screening visit. (6) Individuals who have contracted COVID-19 within the last month as per screening visit. (7) Individuals who have used steroid medications within the last month as per screening visit. (8) Individuals who have been administered immunosuppressants or corticosteroid drugs within the last month as per screening visit. (9) Individuals who have taken medications or health supplements related to immune enhancement within the last month as per screening visit. (10) Individuals with uncontrolled hypertension (systolic blood pressure ≥ 160 mmHg or diastolic blood pressure ≥ 100 mmHg after 10 min of rest). (11) Individuals with uncontrolled diabetes (fasting blood glucose ≥ 160 mg/dL or those who have been taking medication for diabetes in the past 3 months). (12) Individuals suffering from severe gastrointestinal symptoms such as heartburn or indigestion. (13) Individuals who have undergone surgery within the last six months. (14) Individuals with diagnostic medical test results showing: Aspartate aminotransferase (AST), alanine aminotransferase (ALT) > 3 times the reference value; Serum creatinine > 2.0 mg/dL (15) Pregnant or breastfeeding women. (16) Individuals with a history or suspicion of alcohol addiction and drug abuse. (17) Individuals planning to become pregnant during the trial period. (18) Individuals who have experienced hypersensitivity reactions or allergies to ingredients contained in the test/control products of the human clinical trial. (19) Individuals who have participated or plan to participate in another human clinical trial within the last 3 months. (20) Individuals deemed unsuitable for research participation by the trial investigator due to diagnostic medical test results or other reasons.

**Table 2:** General characteristics of the participants.

Variables		Test Group		Placebo Group		Total		*p*- Value ^1^
		*n*	%	*n*	%	*n*	%	
Sex	Male	13	32.5	8	20.0	21	26.3	0.204
	Female	27	67.5	32	80.0	59	73.8	
Age (year)	Mean ± SD	40.85 ± 9.68		39.50 ± 10.19		40.18 ± 9.90		0.545
	Min~Max	23~60		22~55		22~60		
Height (cm)	Mean ± SD	164.87 ± 8.03		163.87 ± 7.12		164.37 ± 7.56		0.558
	Min~Max	151.3~182.3		151.0~183.0		151.0~183.0		
Weight (kg)	Mean ± SD	62.73 ± 13.51		59.82 ± 12.47		61.27 ± 13.01		0.319
	Min~Max	40.0~93.5		44.0~94.0		40.0~94.0		
BMI (kg/m^2^)	Mean ± SD	22.82 ± 3.06		22.13 ± 3.26		22.48 ± 3.16		0.329
	Min~Max	16.2~29.7		17.9~29.5		16.2~29.7		
SBP (mmHg)	Mean ± SD	118.58 ± 13.24		117.60 ± 11.42		118.09 ± 12.30		0.725
	Min~Max	91.0~159.0		95.0~141.0		91.0~159.0		
DBP (mmHg)	Mean ± SD	73.18 ± 9.96		72.68 ± 9.72		72.93 ± 9.78		0.821
	Min~Max	56.0~90.0		49.0~97.0		49.0~97.0		
Pulse (ppm)	Mean ± SD	76.48 ± 9.14		77.15 ± 8.16		76.81 ± 8.62		0.729
	Min~Max	55.0~92.0		60.0~96.0		55.0~96.0		
Alcohol	Drinker	9	22.5	10	25.0	19	23.8	-
	Amount (units/Week)	12.22 ± 7.40		11.04 ± 15.05		11.60 ± 11.75		0.834
Smoke	Smoker	2	5.0	2	5.0	4	5.0	-
	Amount (ea/day)	6.00 ± 1.41		10.50 ± 0.71		8.25 ± 2.75		0.057
	Duration (Year)	37.50 ± 3.54		32.50 ± 17.68		35.00 ± 10.80		0.733

Values are presented as mean ± standard deviation or number (%). ^1^ Analysis by independent *t*-test or Chi-squared test. Abbreviations: BMI, body mass index; SBP, systolic blood pressure; DBP, diastolic blood pressure.

## Data Availability

The datasets generated and/or analyzed during the current study are not publicly available to protect patient confidentiality; however, these are available from the corresponding author upon reasonable request.

## Supplementary Materials

The following supporting information can be downloaded at: https://www.mdpi.com/article/10.3390/nu16060850/s1; Table S1: Compliance test results, Table S2: Intake of major nutrients and minerals in the test and placebo groups, Table S3: Physical activity results, Table S4: Cytokine assay results, and Table S5: Safety outcomes measured by diagnostic laboratory tests.
