# Supplementary material for: Increasing Natural Killer Cell Activity of Mineral Nanomaterial ALP1018 in Healthy Adults: A Randomized, Double-Blind, Placebo Comparative Clinical Trial
Source: Nutrients. 2024 Mar 15;16(6):850. doi: 10.3390/nu16060850 (PMC10976065; doi:10.3390/nu16060850)
Supplement: Supplementary file 1 [file nutrients-16-00850-s001.zip › nutrients-2894656-supplementary.pdf]

Supplementary Materials

Table S1. Compliance test results

Time	Test group	Placebo group	Total
Week 4	93.89±6.27	96.97±5.69	95.45±6.14
Week 8	93.53±7.09	98.23±5.67	95.91±6.79

Values are presented as mean ± standard deviation.

Table S2. Intake of major nutrients and minerals in the Test and placebo groups

Variables	Time Point	Test group	Placebo group	Total	*p*-value[1)]
Energy (kcal)	Baseline	1634.99±392.89	1638.85±564.88	1636.94±484.46	0.972
	Week 4	1813.14±656.72	1742.33±573.72	1777.73±613.53	0.618
	Week 8	1764.20±680.26	1658.16±457.93	1710.49±577.05	0.427
Carbohydrates (g)	Baseline	215.18±67.93	219.95±87.97	217.60±78.24	0.791
	Week 4	213.65±78.88	219.91±88.40	216.78±83.27	0.746
	Week 8	200.53±71.56	212.16±68.01	206.42±69.57	0.467
Lipids (g)	Baseline	54.38±21.69	51.91±21.30	53.13±21.39	0.616
	Week 4	60.03±33.65	57.62±29.08	58.82±31.26	0.738
	Week 8	61.20±41.62	54.19±20.03	57.65±32.50	0.352
Protein (g)	Baseline	64.56±23.01	61.91±22.24	63.22±22.51	0.609
	Week 4	75.22±28.36	72.50±27.05	73.86±27.56	0.670
	Week 8	78.87±32.56	68.37±26.91	73.55±30.10	0.127
Iron (g)	Baseline	12.35±4.20	17.53±16.65	14.97±12.41	0.067
	Week 4	14.33±7.19	14.44±7.95	14.39±7.53	0.948
	Week 8	13.33±6.48	15.70±14.79	14.53±11.46	0.365
Zinc (mg)	Baseline	8.75±3.05	8.10±2.95	8.42±3.00	0.345
	Week 4	10.81±5.62	10.63±5.62	10.72±5.58	0.892
	Week 8	10.29±4.77	9.51±4.44	9.90±4.60	0.460

Values are presented as mean ± standard deviation. [1)] Analyzed by independent t-test.

Table S3. Physical activity results

Variables	Time Point	Test group	Placebo group	*p*-value[1)]
MET (Min/Week)	0 week	2758.24±4211.91	1952.63±2523.83	0.336
	8 week	2672.57±3428.40	2230.56±2259.82	0.525
	p-value[2)]	0.887	0.474	
Work and household activities	0 week	2295.00±5026.81	1260.00±1514.31	0.392
	8 week	1795.56±2852.74	1060.95±1493.97	0.310
	p-value[2)]	0.412	0.792	
Walking and biking to get around	0 week	784.62±540.08	811.43±700.72	0.876
	8 week	1091.33±1319.24	970.30±1025.16	0.684
	p-value[2)]	0.132	0.437	
Leisure time activities	0 week	1056.92±866.01	1160.00±1267.54	0.732
	8 week	1095.38±716.44	1040.00±776.92	0.792
	p-value[2)]	0.728	0.382	
Sitting activities (hour/day)	0 week	5.89±3.44	5.85±3.48	0.951
	8 week	6.34±3.68	5.47±3.37	0.286
	p-value[2)]	0.352	0.488	

MET, metabolic equivalent of task. Values are presented as mean ± standard deviation. [1)] Analyzed by independent t-test. [2)] Analyzed by paired t-test

Table S4. Cytokine assay results

	Time Point	Test group	Placebo group	Total	*p*-value[1)]
TNF-α (pg/mL)	Baseline	7.03±3.18	7.65±3.78	7.34±3.49	0.470
	Week 4	6.74±3.83	7.47±3.57	7.11±3.69	0.348
	Week 8	7.06±3.76	7.87±3.86	7.47±3.81	0.284
IFN-γ (IU/mL)	Baseline	6.30±6.08	7.25±16.22	6.78±12.24	0.447
	Week 4	6.19±5.80	14.60±46.87	10.45±33.66	0.258
	Week 8	6.46±5.72	9.57±29.10	8.04±21.02	0.261
IL-6 (pg/mL)	Baseline	11.39±18.72	5.51±10.69	8.41±15.38	0.168
	Week 4	10.75±17.34	6.57±12.60	8.63±15.18	0.353
	Week 8	11.07±17.48	7.40±16.64	9.21±17.05	0.529

Table S5. Safety outcome measured by diagnostic laboratory test

Variables	Test group	Placebo group	Total	*p*-value[1)]
WBC ($\times10^3$/mm^3)	5.42±1.05	5.15±1.16	5.29±1.11	0.288
RBC ($\times10^6$/mm^3)	4.62±0.35	4.39±0.43	4.50±0.40	**0.012**
Hemoglobin (g/dL)	13.84±1.28	13.35±1.24	13.60±1.28	0.083
Hematocrit (%)	41.85±3.36	40.09±3.40	40.97±3.48	**0.022**
Platelet ($\times10^3$/mm^3)	272.38±63.03	258.43±52.17	265.40±57.91	0.284
Total protein (g/dL)	7.54±0.29	7.42±0.49	7.48±0.40	0.196
Albumin (g/dL)	4.98±0.27	4.86±0.24	4.92±0.26	**0.039**
Glucose (mg/dL)	96.65±10.61	93.48±8.48	95.06±9.67	0.143
Total bilirubin (mg/dL)	0.58±0.31	0.52±0.37	0.55±0.34	0.434
AST (IU/L)	22.00±6.30	21.45±7.46	21.73±6.87	0.723
ALT (IU/L)	16.48±8.69	15.48±11.62	15.98±10.20	0.664
CK (IU/L)	140.55±221.04	113.28±60.74	126.91±161.65	0.456
ALP (IU/L)	56.45±14.14	51.78±14.83	54.11±14.59	0.153
Gamma-GT (IU/L)	26.05±31.44	23.05±35.22	24.55±33.21	0.689
BUN (mg/dL)	13.43±4.02	12.22±3.40	12.82±3.75	0.149
Creatinine (mg/dL)	0.76±0.17	0.73±0.16	0.74±0.16	0.310

Values are presented as mean ± standard deviation. [1)] Analyzed by independent t-test
